# Mucosa-like differentiation of head and neck cancer cells is inducible and drives the epigenetic loss of cell malignancy

**DOI:** 10.1038/s41419-024-07065-y

**Published:** 2024-10-02

**Authors:** Felix Oppel, Sarah Gendreizig, Laura Martinez-Ruiz, Javier Florido, Alba López-Rodríguez, Harkiren Pabla, Lakshna Loganathan, Leonie Hose, Philipp Kühnel, Pascal Schmidt, Matthias Schürmann, Judith Martha Neumann, Flavian Viyof Ful, Lars Uwe Scholtz, Dina Ligum, Frank Brasch, Karsten Niehaus, Germaine Escames, Tobias Busche, Jörn Kalinowski, Peter Goon, Holger Sudhoff

**Affiliations:** 1 Department of Otolaryngology, Head and Neck Surgery, Campus Klinikum Bielefeld Mitte, University Hospital OWL of Bielefeld University, Bielefeld, Germany; 2 Institute of Biotechnology, Biomedical Research Center, Health Sciences Technology Park, University of Granada, Granada, Spain; 3 Department of Physiology, Faculty of Medicine, University of Granada, Granada, Spain; 4 Centro de Investigación Biomédica en Red Fragilidad y Envejecimiento Saludable (CIBERFES), Instituto de Investigación Biosanitaria (Ibs), Granada, San Cecilio University Hospital, Granada, Spain; 5 Center for Biotechnology (CeBiTec), University Hospital OWL of Bielefeld University, Bielefeld, Germany; 6 Proteome and Metabolome Research, Center for Biotechnology (CeBiTec), Faculty of Biology, Bielefeld University, Bielefeld, Germany; 7 Department of Pathology, Klinikum Bielefeld, Bielefeld, Germany; 8 Department of Medicine, Yong Loo Lin School of Medicine, National University of Singapore and National University Health System, Singapore, Singapore

**Keywords:** Cancer stem cells, Oral cancer

## Abstract

Head and neck squamous cell carcinoma (HNSCC) is a highly malignant disease with high death rates that have remained substantially unaltered for decades. Therefore, new treatment approaches are urgently needed. Human papillomavirus-negative tumors harbor areas of terminally differentiated tissue that are characterized by cornification. Dissecting this intrinsic ability of HNSCC cells to irreversibly differentiate into non-malignant cells may have tumor-targeting potential. We modeled the cornification of HNSCC cells in a primary spheroid model and analyzed the mechanisms underlying differentiation by ATAC-seq and RNA-seq. Results were verified by immunofluorescence using human HNSCC tissue of distinct anatomical locations. HNSCC cell differentiation was accompanied by cell adhesion, proliferation stop, diminished tumor-initiating potential in immunodeficient mice, and activation of a wound-healing-associated signaling program. Small promoter accessibility increased despite overall chromatin closure. Differentiating cells upregulated KRT17 and cornification markers. Although KRT17 represents a basal stem cell marker in normal mucosa, we confirm KRT17 to represent an early differentiation marker in HNSCC tissue. Cornification was frequently found surrounding necrotic areas in human tumors, indicating an involvement of pro-inflammatory stimuli. Indeed, inflammatory mediators activated the differentiation program in primary HNSCC cells. In HNSCC tissue, distinct cell differentiation states were found to create a common tissue architecture in normal mucosa and HNSCCs. Our data demonstrate a loss of cell malignancy upon faithful HNSCC cell differentiation, indicating that targeted differentiation approaches may be therapeutically valuable. Moreover, we describe KRT17 to be a candidate biomarker for HNSCC cell differentiation and early tumor detection.

## Introduction

Head and neck squamous cell carcinoma (HNSCC) is a highly malignant disease that represents the 6th most common type of cancer in the world. Five-year survival rates of about 50% have remained unchanged for decades [1, 2]. Despite recent advances in molecular targeting and immunotherapy, the prognosis of affected patients remains devastating. Even though new approaches in immunotherapy and molecular therapy exist, the standard of care for HNSCC remains surgery, cytotoxic chemotherapy, and radiotherapy. These treatments show limited tumor-specificity and thus frequently damage vital structures in the head and neck area, leading to high morbidity even in long-term survivors [3]. Hence, new tumor-specific therapy approaches are urgently needed.

Squamous cell carcinomas are characterized by cornified areas detectable by histopathology. Cornification represents the normal differentiation path of keratinocytes in the epidermis and the oral mucosa and ensures the formation of a protective barrier on our body’s surface [4]. In the malignant context however, “cell differentiation” is defined by how closely a tumor cell resembles its original tissue, but little is known about the potential of long-term repopulating tumor cells to terminally differentiate and lose their malignancy due to epigenetic changes like chromatin remodeling processes. It is believed that the malignant cell state may prevent terminal differentiation [5] or causes a plastic differentiation behavior with mixed or reversible differentiation phenotypes [6–9]. In this study, we have modeled HNSCC cell differentiation, describe differentiation markers, show close structural similarities to the normal tissue counterpart, and established an initial protocol to trigger this process. This may provide a rationale for the development of differentiation therapies targeting HNSCC cell malignancy.

## Materials/subjects and methods

### Human material and cell culture

Primary head and neck cancer tissue was obtained from medically indicated surgeries with informed consent of the patients, according to the declaration of Helsinki, and as approved by the ethics committee of the Ruhr-University Bochum (AZ 2018-397_3), as reported previously [10, 11]. Primary cells were cultured in PneumaCult™-Ex Plus Medium, referred to as stem cell medium (SCM, #05041, Stemcell Technologies, Vancouver, Canada) supplemented as described previously [11]. Adherent cells and spheroids were detached using Accutase (Capricorn Scientific, Ebsdorfergrund, Germany). Cells were differentiated in cardiac fibroblast medium (CFM, 316K-500, Cell Applications, San Diego, CA, USA) supplemented with 1% 200 mM l-glutamine, 1% 100× penicillin/streptomycin, and 1% 250 µg/mL Amphotericin B solution (all three Capricorn Scientific). Dulbecco’s Modified Eagle Medium (DMEM, DMEM-HXA, Capricorn Scientific) was supplemented with 10% Fetal Bovine Serum Advanced (FBS Advanced, FBS-11A, Capricorn Scientific), l-glutamine, Penicillin/Streptomycin, and Amphotericin B. Cells received fresh medium twice a week. Cells were analyzed for proliferation, and cornification, and treated with drugs and cytokines as outlined in supplementary methods.

### Mouse xenograft tumor models

All animal experiments were conducted as approved by the Institutional Animal Care and Use Committee of the University of Granada (procedures 12/07/2019/128) in accordance with the European Convention for the Protection of Vertebrate Animals used for Experimental and Other Scientific Purposes (CETS #123) and Spanish law (R.D. 53/2013).

NSG mice (NOD.Cg-Prkdcscid Il2rgtm1Wjl/SzJ, The Jackson Laboratory, Bar Harbor, ME, USA) were housed in appropriate sterile filter-capped cages with sterile food and water ad libitum. 1 × 10^6^ cells were transplanted subcutaneously as described previously [12], with differentiated and undifferentiated cell populations being injected side-by-side into the left and right flank of each mouse as performed in a previous study [8]. Animals were monitored for tumor development twice a week. Tumor sizes were measured using a vernier caliper and tumor volume was calculated by the formula (length × width^2^)/2. When the first tumor of each population reached the legal size limit, all mice of that experiment were measured and sacrificed. Tumors were harvested and fixed in 4% paraformaldehyde (PFA, 2.529.311.214, PanReac) for 24 h. The fixed samples were embedded in paraffin following standard protocols.

### Histopathology analysis of HNSCC tissue

Paraffin-embedded tissue was sectioned to 2 µm thickness using a sliding HM430 microtome (Zeiss, Oberkochen, Germany). Hematoxylin/Eosin (HE) staining was performed using standard protocols in a linear COT 20 tissue stainer (MEDITE, Burgdorf, Germany). HE-stained sections were analyze by a senior pathologist (F.B.) to compare patient characteristics between original tumor and xenograft tumor models. To quantify keratinized areas in human tumor tissue every HE-stained section was subdivided into three random views and analyzed using an Axio Lab.1 microscope (Zeiss) and DISKUS software (Hilgers, Königswinter, Germany). Per view, the total area displaying malignant histology was determined in mm^2^. Next, the keratinized area within the tumor compartment was determined to calculate the proportional extent of cornification using Microsoft Excel.

### Indirect Immunofluorescence of HNSCC cells and tissue

Indirect Immunofluorescence (IF) of HNSCC cell cultures was performed as described previously [13]. In brief, cells grown on coverslips were fixated using ice-cold 4% paraformaldehyde (PFA) in PBS and permeabilized by a 5 min incubation in ice-cold permeabilization buffer (0.1% Citrate, 0.1% Triton X-100). DNA was stained with DAPI (Sigma Aldrich, St. Louis, MO, USA) using 2 µg/ml diluted in blocking buffer (PBS + 0.1% bovine serum albumin, Capricorn Scientific). Subsequently, the primary antibody diluted in blocking buffer was applied directly onto the coverslip followed by a 1.5 h incubation. After three washing steps with blocking buffer, the cells were incubated for 1.5 h with the secondary antibodies diluted in blocking buffer. Finally, the samples were washed 1× in blocking buffer, 1× in PBS, and 1× in H_2_Odd and mounted on a glass slide using an aqueous mounting medium (Santa Cruz Biotechnology, Dallas, TX, United States). Tumor tissue of patients and xenograft tumor models were analyzed by IF as established in prior studies [8]. Briefly, paraffin-embedded tissue sections were de-paraffinized and rehydrated according to standard procedures. Next, the slides were cooked 4× in 10 mmol citric acid/sodium citrate unmasking buffer (pH 6; 18% citric acid, 82% citrate) using a microwave oven, each followed by a 15 min incubation. After that, the slides were blocked for 15 min in blocking buffer. The subsequent staining procedure with DAPI and antibodies was performed as described for fixated cells above, with the staining solutions being applied onto a pan-pen surrounded area of the tissue section. All samples were imaged using an LSM780 confocal microscope (Zeiss) and ZEN software (Zeiss). Utilized antibodies are listed in Supplementary Methods.

### RNA isolation and real-time quantitative PCR

Total RNA was isolated from cultured cells using innuPREP DNA/RNA Mini Kit (Analytik Jena, Jena, Germany) as recommended by the manufacturer’s protocol. RNA quality was determined with a BioDrop Duo+ spectral photometer (Biochrom, Holliston, USA). Real-time quantitative PCR (RT-qPCR) was conducted as described previously [11] using the following primers:

GAPDH-forward: CTGCACCACCAACTGCTTAG,

GAPDH-reverse: GTCTTCTGGGTGGCAGTGAT,

ITGB1-forward: GACGCCGCGCGGAAAAG,

ITGB1-reverse: TCTGGAGGGCAACCCTTCTT,

KRT17-forward: CCTCAATGACCAACACTGAGC,

KRT17-reverse: GTCTCAAACTTGGTGCGGAA,

SPRR3-forward: TGCACAGCAGGTCCAGCA,

SPRR3-reverse: GGCTCCTTGGTTGTGGGAA.

### Multi-omics analysis of HNSCC cell differentiation

Differentiated and undifferentiated HNSCC cell populations were analyzed using ATAC-seq and RNA-seq. Both types of cultivation media were examined for their components through proteomics and metabolomics. These methods are detailed in the Supplementary Methods section.

### Single-cell cloning assay

Cells were detached and seeded into U-bottom 96-wells at a density of approximately 1 cell/well in 100 µl volume of the desired medium. Plates were centrifuged and all wells were examined in a light microscope (Leica, Wetzlar, Germany) to identify wells containing single cells. These wells were examined after 21 days for survival and proliferation. Cells received fresh medium once a week. Obtained data were processed in Microsoft Excel (Microsoft Corporation, Redmond, WA, USA) and visualized using GraphPad Prism software (GraphPad Software Inc., San Diego, CA, USA).

## Results

### Primary HNSCC spheroid models

**Fig. 1:**
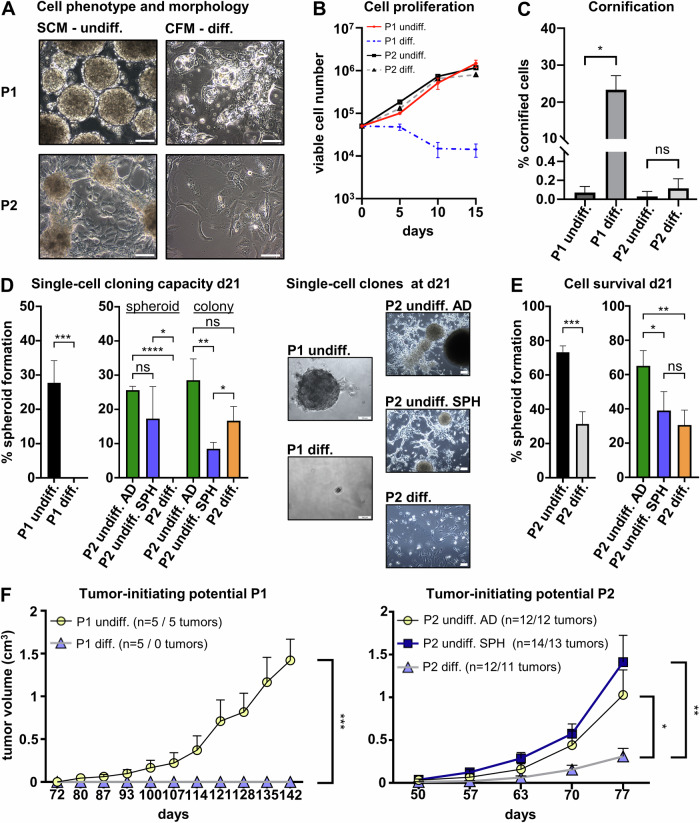
A HNSCC differentiation model reveals a loss of cell malignancy. **A** Cells of P1 grow as spheroids in SCM (undifferentiated), whereas P2 cultures display a loosely adherent cell layer that forms spheroids and releases them into the culture medium. Treatment of SCM cultures with differentiation medium (CFM) results in the formation of an adherent layer of enlarged cells with irregular shapes. P1 cells show in CFM show cell inclusions and bubble-like structures reminiscent of cornification. **B** CFM-treated cells of P1 lose proliferation, whereas cells of P2 continue to expand. **C** Cornification assay reveals cornified envelope formation in CFM-treated cells of P1 only; *p = 0.0005; ns not significant. **D** Single-cell cloning assay for 21 days (d21) confirms efficient self-renewal and clonal repopulation capacity of P1 spheroid cells which was totally abolished in CFM. Adherent and spheroid cells of P2 in SCM were equally able to form spheroids from single cells. Spheroid cells in SCM showed a reduced tendency to form adherent colonies from single cells. Single cells of P2 in CFM could not form spheroids; *p < 0.05; **p < 0.01; ***p < 0.005; ****p < 0.0001; ns not significant. Examples of representative clonal cultures at day 21 (d21) are displayed. **E** Treatment with differentiation medium for 21 days reduces the survival of single-cell clones of P1 and P2; *p < 0.05; **p < 0.01; ***p < 0.001; ns not significant. **F** Subcutaneous transplantation of undifferentiated and differentiated populations into immunodeficient NSG mice showed diminished tumor-initiating cell potential of CFM-treated cells of P1 and P2. No significant differences were observed between adherent (AD) and spheroid (SPH) cells of P2 cultured in SCM; *p < 0.05; **p < 0.005; ***p < 0.0005. *p*-values were determined by student’s t-test at the endpoint of the experiment.

The process of keratinization is a characteristic of squamous cell carcinomas. To study this differentiation path in HNSCC, we employed the primary spheroid model S18 derived from human papillomavirus (HPV)-negative oropharyngeal HNSCC tissue of a previous study [11], further referred to as patient 1 (P1). Moreover, we established a primary cell culture from a xenograft tumor model of another study [10] which was derived from a second oropharyngeal HPV-negative HNSCC (P2; Supplementary Table S1). The xenograft tumors of P2 closely resembled the original tumor in histology (Supplementary Fig. S1A) and adherent cells were isolated from the xenograft tumor tissue that could be expanded for more than 20 passages in stem cell medium (SCM) without phenotypic changes (Supplementary Fig. S1B). These cells formed a spindle-like network of adherent cells that released spheroids into the supernatant (Fig. 1A, Supplementary Fig. S1B). After spheroids were harvested from the supernatant, detached, and re-plated into normal flasks, cells regenerated the adherent spheroid-forming cell layer (Supplementary Fig. S1C), indicating that the spheroid cells can repopulate the original cell culture.

### HNSCC differentiation attenuates cell malignancy

A known protocol to interfere with tumor stem cell self-renewal and to induce differentiation is to change from serum-free culture conditions to a serum-containing medium [8, 9, 14]. Cardiac fibroblast medium (CFM), a serum-containing medium, displayed a striking differentiating effect on HNSCC cells (Fig. 1A). Upon treatment with this medium, P1 cells fully adhered to the culture dish, displaying strongly increased cell sizes with pleomorphic cell shapes (Fig. 1A) and lost proliferation (Fig. 1B). P2 xenograft tumor cells also adhered and acquired enlarged pleomorphic cell bodies (Fig. 1A) but continued to proliferate (Fig. 1B). CFM-cultured cells of P1 developed thickened cell envelopes and cytoplasmic inclusions reminiscent of lamellar bodies, indicative of cornification. Indeed, CFM-cultured P1 cells contained approximately 200x more cornified envelopes than control cells (Fig. 1C). This was insignificant in P2. SCM-cultured cells of P1 expanded from single cells to whole spheroids/colonies with an efficiency of about 30%, which was entirely abolished in CFM. P2 cells in CFM lost the spheroid-forming capacity but grew as adherent colonies, whereas single-cell clones of adherent and spheroid SCM cells both reestablished the mixed adherent/spheroid culture (Fig. 1D). Moreover, CFM treatment compromised the survival of P1 and P2 clones (Fig. 1E). Transplantation of P1 and P2 cells into immunodeficient mice resulted in efficient xenograft tumor formation from SCM cells, whereas CFM populations displayed strongly reduced (P2) or even annihilated (P1) tumor-initiating cell (TIC) capacity (Fig. 1F). Within the SCM culture of P2, adherent and spheroid cells were equally able to initiate tumors. In P1, histopathology analysis also revealed cystic tissue architecture with large areas of necrosis that more closely resembled lymph node metastases than the original tumor (Supplementary Fig. S2A). For P2, a similar cystic tissue architecture of xenograft tumors was observed (Supplementary Fig. S2B). CFM and SCM tumors of P2 matched in histology with insignificantly different extents of tissue cornification (Supplementary Fig. 2C) indicating that remaining TIC after differentiation treatment retained their imprinted differentiation behavior.

We conclude that CFM treatment induces functional and phenotypic differentiation in HNSCC cells, which will further be referred to as differentiation medium (diff. medium). Moreover, these results show that the differentiation of primary HNSCC cells strongly reduces their malignant properties and can cause a phenotype reflecting normal corneocytes.

### HNSCC cell differentiation is associated with chromatin remodeling

**Fig. 2:**
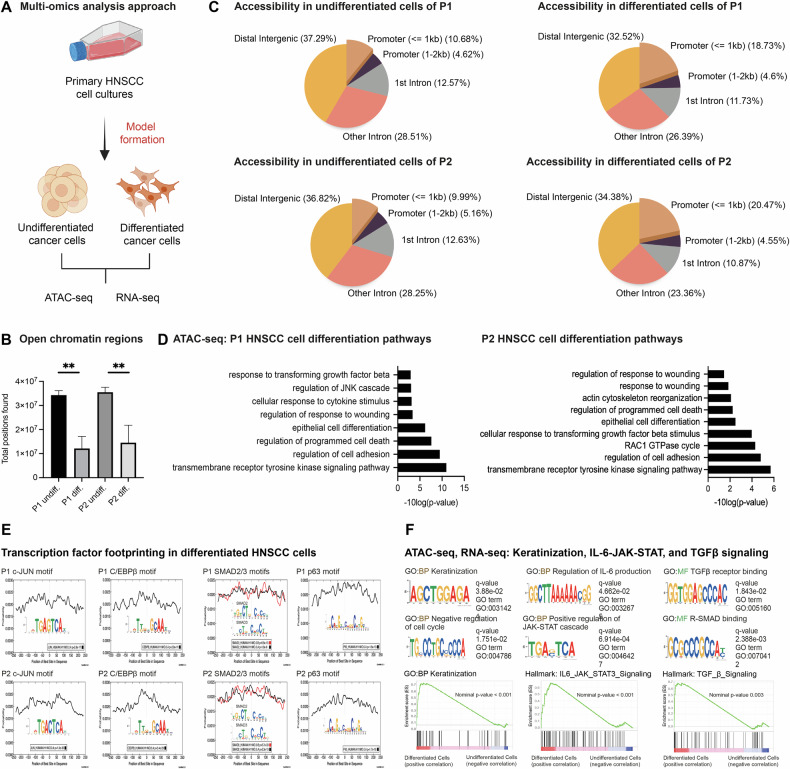
ATAC-seq identifies differentiation-associated signaling in HNSCC cells. **A** Overview of the omics-approaches to identify the changes underlying tumor cell cornification in HNSCC. **B** Genomic annotation of the OCRs in the cancer cells from both HNSCC patients showed a decrease in accessible areas upon differentiation (**p < 0.01) with technical replicates (*n* = 3) and a statistical cut-off q-value of 0.05 for peak calling. **C** Despite an overall chromatin closure, the proportion of small open promoters (<1 kb) increased. **D** GO terms display the relevant signaling pathways linked to the differentially called OCRs of differentiated cells of both patients with Fisher’s exact test. **E** Transcription factor footprinting identified p63, c-JUN, C/EBPβ, and SMAD2/3 motifs in differentiating cancer cells. **F** Relevant pathways of HNSCC differentiation are Keratinization, IL6-JAK/STAT3, and TGFβ signaling, identified by gene ontology motif analysis (GOMo, ATAC-seq) and GSEA (RNA-seq); GO:BP Gene Ontology Biological Process, GO:MF Gene Ontology Molecular Function.

We performed ATAC-seq and RNA-seq to analyze the changes associated with tumor cell differentiation (Fig. 2A). ATAC-seq confirmed a reduction of open chromatin regions (OCRs) in the genome upon differentiation by 64.6% in P1 cells and by 59.08% in P2 cells (Fig. 2B, Supplementary Table S2), indicating closure of the genome reminiscent of stem cell differentiation in other systems [15–17]. Despite this net loss of chromatin accessibility, the proportion of open small promoters (<1 kb) increased 1.75-fold in differentiated P1 cells and 2.05-fold in P2 cells. Larger promoter regions (>1 kb) were proportionally not affected (Fig. 2C, Supplementary Table S3). In both patients, ATAC-seq displayed that the differentiation of HNSCC cells made OCRs accessible that are associated with epithelial cell differentiation, cell adhesion, and stress-associated pathways, which included the regulation of response to wounding, programmed cell death, response to TGFβ, regulation of JNK cascade, and transmembrane receptor tyrosine kinase signaling (Fig. 2D, Supplementary Tables S4–S7). Transcription factor footprinting unraveled the activation of TGFβ signaling via R-SMAD proteins SMAD2/3/4, activator protein-1 (AP-1) signaling via c-JUN, JAK/STAT3 signaling, interleukin 6 (IL6), and the pro-inflammatory transcription factor C/EBPβ (Fig. 2E).

**Fig. 3:**
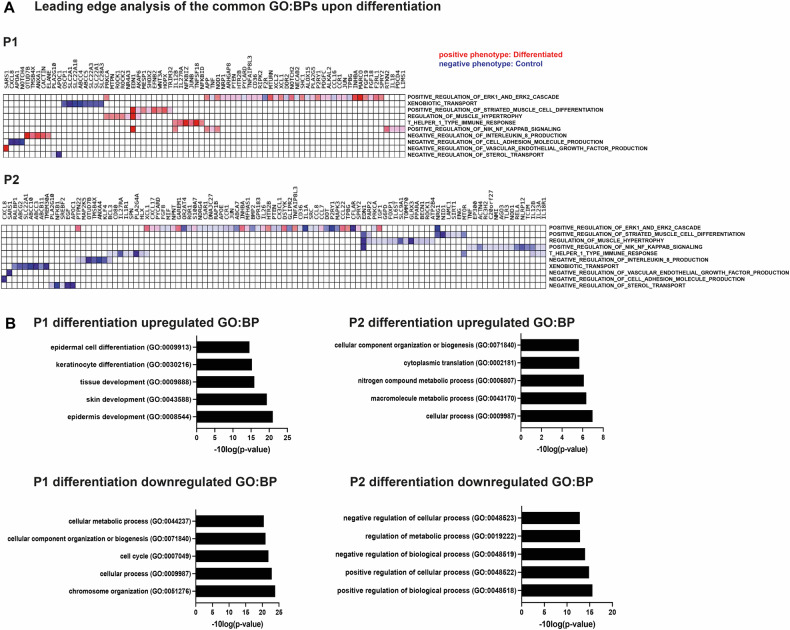
RNA-seq analysis reveals global expression changes upon HNSCC cell differentiation. **A** The leading edge analysis highlights driver genes based on the Gene Ontology Biological Process (GO: BP) that the differentiation models of both patients have in common, with a cut-off *p*-value of 0.05. **B** Enrichment analysis shows the top five significant transcriptome changes with associated Process GO:BP pathways. Three technical replicates were used (*n* = 3) with a statistical cut-off *p*-value of 0.05.

RNA-seq revealed hundreds of differentially expressed transcripts upon HNSCC cell differentiation (Supplementary Tables S8–S11). Gene Set Enrichment Analysis (GSEA) confirmed the results of ATAC-seq identifying keratinization, IL6-JAK-STAT, and TGFβ to be associated with HNSCC cell differentiation (Fig. 2F). On protein level, the activation of signaling via TGFβ, c-JUN, STAT3, and C/EBPβ was also confirmed in adherent cells of P1 and P2 (Supplementary Fig. S3). Leading edge analysis identified potential driver genes of 10 common pathways in both differentiation models of P1 and P2 (Fig. 3A). Genes related to NF-κB signaling are positively enriched in differentiated P1 cells and negatively enriched in P2, with *EDN1* being the most enriched gene. Genes associated with xenobiotic transport were negatively enriched in differentiated cells of both patients. Moreover, both patients tendentially showed an enrichment of genes implicated in the ERK1 and ERK2 signaling cascades. However, most driver genes of the identified pathways differed in both patients (Fig. 3A).

In differentiated cells of P1, Gene Ontology (GO) analysis indicated upregulated pathways associated with the differentiation of keratinocytes and skin development (Fig. 3B, Supplementary Tables S12–S15).

As the components of both media used here either supported a stem-like phenotype (SCM) or a differentiated cell state (diff. medium), ESI-LC/MS analysis was used to reveal the proteins contained in both media (Supplementary Tables S16 and S17). The differentiation medium contained a complex protein ensemble of 132 exclusive analytes, while the SCM medium contained a less complex mixture with 14 exclusive proteins. The proteins in the differentiation medium showed enrichment in signaling pathways via p53 and NOTCH1 and in biological processes like wound healing, skin development, epithelium development, and tissue development (Supplementary Fig. S4). This indicates that the differentiation medium itself contains skin components and that these may be responsible for the differentiation observed in our model. A metabolomic GC-MS analysis of the media revealed that the differentiation medium contained significantly fewer amino acids than SCM (Supplementary Table S18).

### High expression of KRT17 and SPRR3 associates with HNSCC differentiation

**Fig. 4:**
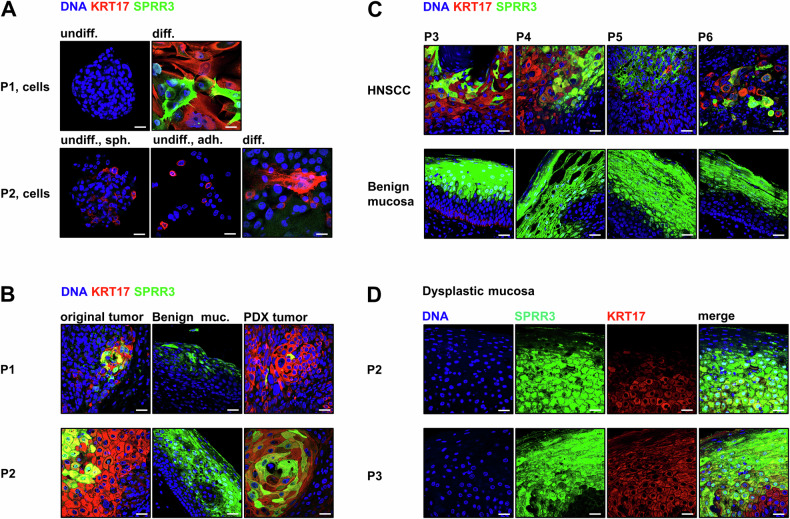
KRT17 expression indicates premature differentiation stages in HNSCC tissue and dysplastic mucosa. **A** P1 cells upregulate KRT17 and late cornification marker SPRR3 upon treatment with differentiation medium (CFM) while undifferentiated spheroid cells in stem cell medium (SCM) stain negative for this marker. In P2, KRT17 expression is restricted to a subpopulation in both media which was also observed in adherent (adh.) and spheroid (sph.) cells in SCM. **B** KRT17 was strongly expressed around SPRR3+ patches in the original patient’s HNSCC tissue and xenograft tumors in immunodeficient mice of P1 and P2, but not in normal mucosa of those patients. KRT17 seems to precede SPRR3 expression in human HNSCC tissue. **C** The tumor-specific association of KRT17 and cornification indicated by SPRR3 was reproduced in four additional patients (P3-P6). In benign mucosa of the same individuals KRT17 was barely visible in the basal layer only or not detectable, when imaged using the same instrument settings. **D** Dysplastic mucosa attached to the tumor tissue of P2 and P3 showed a more abundant expression of KRT17 compared to normal mucosa spreading from the basal layer into the SPRR3+ zone; staining by indirect immunofluorescence (IF); scale bars = 10 µm.

As cornification represented the main reaction of P1 cells to differentiating culture conditions, we searched for markers of this process. The cornification marker *SPRR3* was 9.06-fold more accessible upon differentiation in P1 and was upregulated 94.09-fold (Supplementary Tables S4 and S8). *KRT17* showed 14.59-fold increased accessibility, was upregulated 59.29-fold, and displayed a striking accessibility peak only in differentiated P1 cells (Supplementary Fig. S5). In P2, *KRT17* expression increased 2.8-fold (Supplementary Table S12). On protein level, IF analysis confirmed a strong upregulation of KRT17 and SPRR3 in diff. medium-treated P1 cells (Fig. 4A). P1 spheroids stained predominantly negative for these markers. In P2, KRT17 was expressed by a subpopulation of cells under any condition, whereas SPRR3-positive cornified envelopes were not detected (Fig. 4B). To further investigate KRT17 and SPRR3 as markers of HNSCC differentiation, we stained the original tumor tissue of P1 and P2 and HPV-negative tumor tissue of four additional patients with oropharyngeal and hypopharyngeal tumor location (P3-6, Supplementary Table S1). Normal mucosa attached to the surgically removed tumor piece showed KRT17 expression only in basal cells, which was downregulated upon tissue maturation. SPRR3 displayed nuclear localization starting midway in the stratum spinosum, followed by the formation of an SPRR3+ cell envelope towards the outer mucosal layers (Fig. 4B, C). In contrast, KRT17 appeared to precede SPRR3 in the HNSCC tissue of all examined patients surrounding keratinized areas, as described previously [18], and to co-localize with SPRR3 towards the core of keratin pearls (Fig. 4B, C). This indicates that KRT17 is a tumor-specific differentiation marker. P2 and P3 displayed hyperplastic/dysplastic tissue adjacent to the tumor. Interestingly, in both cases the KRT17 expression pattern reflected the tumor and not the normal mucosa (Fig. 4D), highlighting the reversal of KRT17 from a basal stem cell marker to a cornification-associated differentiation-indicating protein to be an early event in HNSCC development. Thus, these data highlight KRT17 as a differentiation marker in HPV-negative HNSCCs and propose it as possible biomarker for early tumor detection.

### HNSCC differentiation can be triggered by inflammatory mediators

**Fig. 5:**
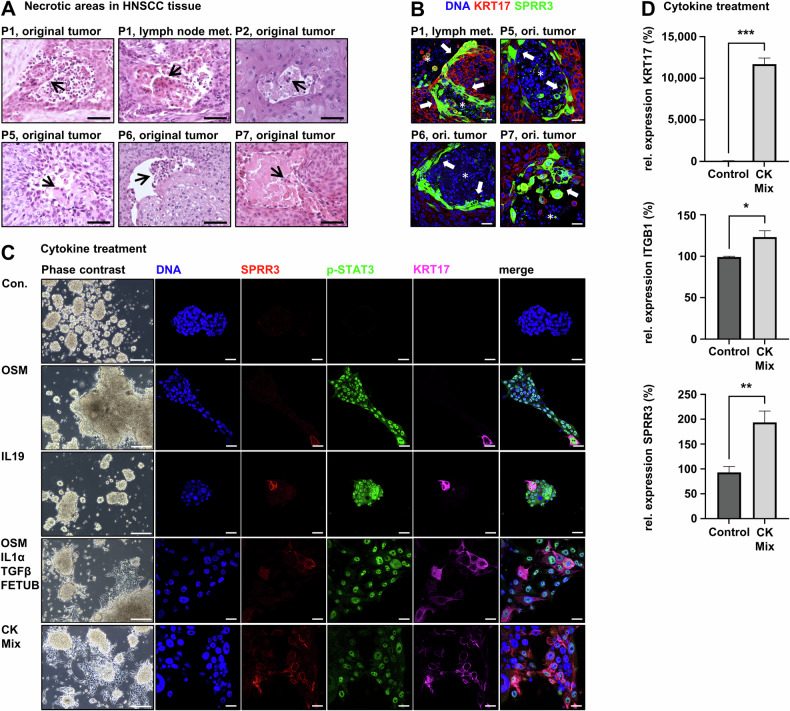
Inflammatory cytokines trigger differentiation in HNSCC cells. **A** HNSCC tissue shows cornified cells at the interface between vital tumor tissue and necrotic areas and dead cornified cells in the necrotic cavity (arrows); scale bars = 50 µm. **B** IF analysis confirms the upregulation of cornification marker SPRR3 at the edge of necrotic areas (arrows); necrotic tissue is indicated by *; scale bars = 10 µm. **C** Treatment of undifferentiated HNSCC cells with combinations of inflammatory cytokines results in cell adhesion, activation of signaling through phospho-STAT3-Y705 (pSTAT3), and upregulation of differentiation marker KRT17 and cornification marker SPRR3 compared to the untreated control (Con.). Single cytokines like IL19 and OSM were able to induce this differentiation phenotype, but higher numbers of cytokines in combination worked increasingly effective; cytokine mix (CK Mix) = EGF, IL1α, IL1β, IL6, IL17A, IL22, TGFβ, OSM, and TNFα. Cells were treated for 5 days in SCM without hydrocortisone; cytokine concentration = 30 ng/ml; scale bars phase contrast = 200 µm; scale bars IF = 10 µm. **D** RT-qPCR shows increased relative expression of adhesion marker ITGB1, KRT17, and SPRR3 compared to the untreated control. Cells were treated with CK Mix for 3 days in SCM without hydrocortisone; cytokine concentration = 30 ng/ml; *p < 0.01; **p < 0.005; ***p < 0.0001, determined by student’s t-test.

KRT17 is described to be upregulated in keratinocytes affected by psoriasis, a chronic inflammatory disease mechanistically resembling wound healing [19]. Interestingly, psoriasis is driven by the activation of many signaling pathways and transcription factors, including STAT3, C/EBPβ, and AP-1, triggered by damage- or inflammation-associated molecules released from dying keratinocytes and invading immune cells. Thus, we investigated whether there is a mechanistic connection between inflammatory signaling and HNSCC cornification. In histology, we noticed that keratinization of the original tumor tissue of P1 was more intense in lymph node metastases, which generally show a more (pseudo-)cystic growth pattern than primary tumors in HNSCC with cysts containing keratin and cellular debris [20]. P1 primary tumor tissue showed only small patches of keratinization, whereas lymph node metastasis tissue displayed larger differentiated areas predominantly surrounding necrotic areas, which was also observed in tumor tissue of other patients (Fig. 5A). IF staining revealed the expression of cornification marker SPRR3 precisely at the interface between necrotic cysts and the vital tumor tissue (Fig. 5B). Thus, we hypothesized that cornification might be triggered by inflammatory signaling induced by the necrotic environment, as cytokines influence the differentiation of keratinocytes in normal skin and psoriasis [19, 21]. Thus, we treated P1 cells with the described cytokines in SCM. Inflammatory cytokines induced a differentiation phenotype in HNSCC spheroid cells (Fig. 5C). Most effectively, the combination of EGF, IL1α, IL1β, IL6, IL17A, IL22, TGFβ, oncostatin-M (OSM), and TNFα induced adhesion and upregulated the expression of adhesion marker ITGB1 and the differentiation markers KRT17 and SPRR3 (Fig. 5C, D). These data show that differentiation can be imposed on HNSCC cells by the targeted application of inflammatory mediators.

### HNSCC tissue keratinization recapitulates normal mucosa architecture

**Fig. 6:**
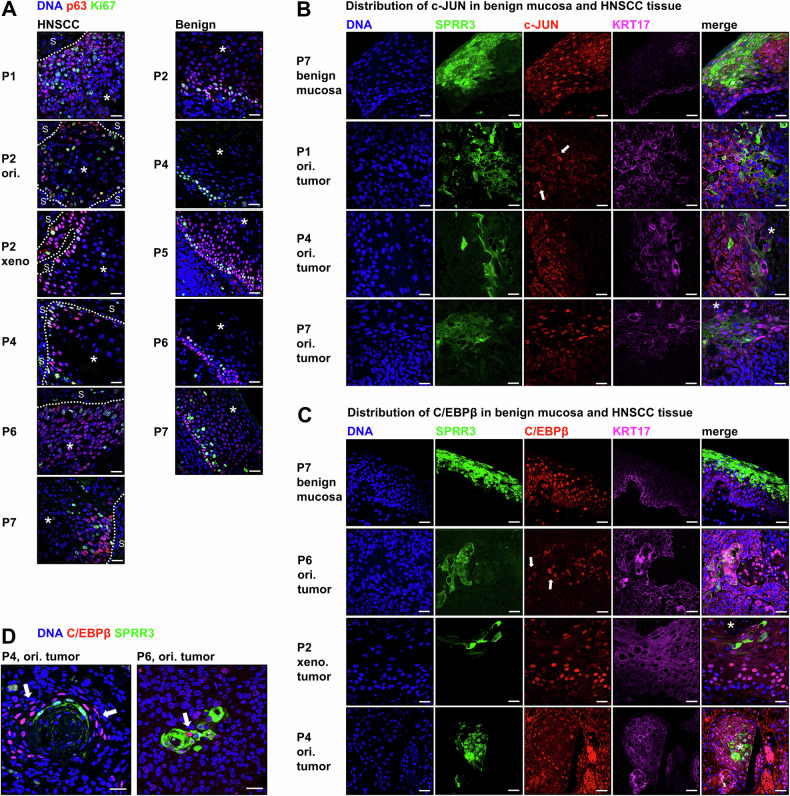
HNSCC tissue architecture resembles benign mucosa maturation. **A** Expression of basal keratinocyte marker p63 [22] and proliferation marker Ki67 were analyzed by indirect immunofluorescence. In benign mucosa, nuclear p63 is observed in the basal layer and in the lower stratum spinosum with Ki67+ cells located in a small layer above the basal cells. In HNSCC, a zone of cells positive for p63 and/or Ki67 is located at the tumor-stroma interface (dashed line, S = stroma) and both markers are downregulated towards the area of tumor differentiation (indicated by *). For example, P1 tumor tissue is largely undifferentiated, poorly organized, and shows abundant p63 and Ki67 expression which decreases surrounding the necrotic area. In contrast, HNSCCs of P2 and P4 show higher differentiation and p63/Ki67 expression is restricted to a small layer of cells. HNSCC activates signaling via transcription factors c-JUN (**B**) and C/EBPβ (**C**). Representative examples are shown for each marker: one benign mucosa sample, one poorly differentiated HNSCC (P1 or P6), and two higher differentiated HNSCCs (P2 and P4 or P4 and P7). Highly organized tumors and benign mucosa display a layer of cells with nuclear c-JUN or C/EBPβ right above the p63/Ki67+ layer detected in (**A**). In these samples, basal cells in benign tissue and cells at the tumor-stroma interface in HNSCC show cytoplasmic location of these markers. Poorly organized tumors show fewer cells with nuclear c-JUN or C/EBPβ expression (arrows) which were mixed in between the SPRR3+ differentiated tumor cells indicating a perturbated layer organization. **D** Large (P4) and small (P6) areas of cornification harbor a zone of active signaling close to or within the SPRR3+ zone (indicated by nuclear C/EBPβ, arrows). Xeno. tumor = xenograft tumor tissue in immunodeficient mice; ori. tumor = original tumor tissue; dyspl. = dysplastic; scale bars = 10 µm.

Above, we identify an inflammatory reaction of HNSCC cells upon differentiation that can be triggered by cytokines. To verify our results in a larger set of patients, we examined the activity of these pathways utilizing indirect immunofluorescence in the original tumor tissue of P1, P2, and five additional patients (P3-P7) with HPV-negative HNSCCs originating from oropharyngeal, hypopharyngeal, and laryngeal location (Supplementary Table S1, Supplementary Fig. S6), as well as the xenograft tumor tissue of P1 and P2. This set of HNSCC tissue samples contained samples with abundant keratin pearls (P2–P5, P7) and others with rather single-cell keratinization (P1, P6, Supplementary Figs. S2 and S6). IF staining for c-JUN, C/EBPβ, basal stem-cell marker p63 [22], and proliferation marker Ki67 in combination with the differentiation markers KRT17 and SPRR3 revealed four different zones of marker expression in normal mucosa (Fig. 6A–D):

(1) p63+/KRT17+ basal cells. (2) Cells in the stratum spinosum directly above the basal layer express Ki67 and p63 but lose KRT17 expression and activate c-JUN and C/EBPβ, as evidenced by nuclear localization of these effectors. About midway in this zone of intense signaling, cells start to express SPRR3. (3) Towards the stratum granulosum, SPRR3 changes to a cytoplasmic location as part of the cornified envelope. Cell signaling is lost gradually as cells shut down viable processes during certification. (4) In the stratum corneum, cells display pyknotic nuclei and stain negative for any marker, presumably due to epitope masking by transglutaminase activity.

When we compared this normal mucosal structure to keratinized areas in HNSCC, we observed that these zones were reflected in the tumor tissue (Fig. 6A–D). Tumors with intense cornification showed a zone comprised of relatively small cells at the stromal interface lacking expression of c-JUN and C/EBPβ but stained positive for Ki67 and/or p63. In closer proximity to keratin pearls, cells become larger, with more cytoplasm and nuclei appearing hollow and spotted, reminiscent of heterochromatin formation. These cells predominantly stain positive for c-JUN and C/EBPβ without p63 and Ki67, indicating loss of proliferation (Fig. 6A–C). KRT17 and SPRR3 are gradually upregulated in this zone. At the interface between this zone and destained fully keratinized tumor cells with pyknotic nuclei, cells display variable morphology and strong SPRR3 expression. Interestingly, in tissues with a low tendency to cornify (P1 and P6) these distinct differentiation states were also found. Unlike their organization into sequential layers in normal mucosa and well-differentiated tumor tissue, the distinct “layers” in low-differentiated HNSCC tissue consisted of few or single cells only (Fig. 6D).

## Discussion

### HNSCC cells recapitulate controlled stem cell differentiation

In this study, we modeled the transition of undifferentiated tumor-initiating cells into tumor corneocytes. Cornification is the normal differentiation path in the oral mucosa, which ultimately leads to cell death [4]. As dead cells cannot de-differentiate and return to a basal keratinocyte state, we assume that this differentiation is terminal. In benign and malignant tissue, dead corneocytes thus represent a common differentiation end product and, as these cells cannot exert malignant behavior, we consider them as benign.

In HNSCC cells, we show that this differentiation was accompanied by an overall genome closure similar to stem cell differentiation in other systems [15–17]. Thus, the state of chromatin accessibility upon tumor differentiation was successfully altered, observing a significantly increased proportional accessibility of small promoter regions, which may relate to the ongoing expression of housekeeping genes with simple promoters lacking control by distal enhancers [23]. This represents a remodeling process from euchromatin to heterochromatin, forming the differentiated state [24]. It is known that TIC formation and maintenance can be controlled epigenetically [25], but little is described about which epigenetic mechanisms allow TICs to differentiate into bulk tumor cells. Single-cell RNA-seq revealed that TICs differentiate similar to normal stem cells but in a skewed way acquiring irregular phenotypes [6]. Compared to single-cell techniques using human tissue, the bulk analysis in our model allows a simultaneous analysis of the malignancy of distinct cell populations. In this way, we show a clear loss/reduction of clonal repopulation and tumor-initiating capacity upon forced tumor cell differentiation, highlighting the targeted induction of cornification as potential therapy approach.

### HNSCC tissue retains the differentiation program of normal mucosa

Here, we identify the activation of a wound-healing-like signaling via TGFβ, JNK/AP1, IL6/JAK/STAT3, and C/EBPβ to be an early event in the differentiation process of benign and malignant tissue. This signaling program resembles wound healing and psoriasis [19], and these pathways are well-described as oncogenic in specific contexts, predominantly c-JUN [26], IL6/JAK/STAT3 [27], and TGFβ [28]. C/EBPβ seems to exert an isoform-specific oncogenic role in breast cancer [29]. However, in certain malignancies, these molecules can exert anti-tumor properties in a context-dependent and tumor-stage-dependent manner as well. TGFβ is known to function as a tumor suppressor in early-stage tumor development and as an oncogene in later stages [28]. Other studies implicate c-JUN [30] and STAT3 [26] in tumor suppressive functions, too. In HNSCC, there is no proof for their direct implication in malignant functions, even though high expression of c-JUN was shown to correlate with poor overall survival [31]. Our data show that c-JUN signaling is repressed in p63 + /Ki67+ tumor stem-like cells and rather activated after the loss of the proliferation marker Ki67, indicating a function in differentiation.

**Fig. 7:**
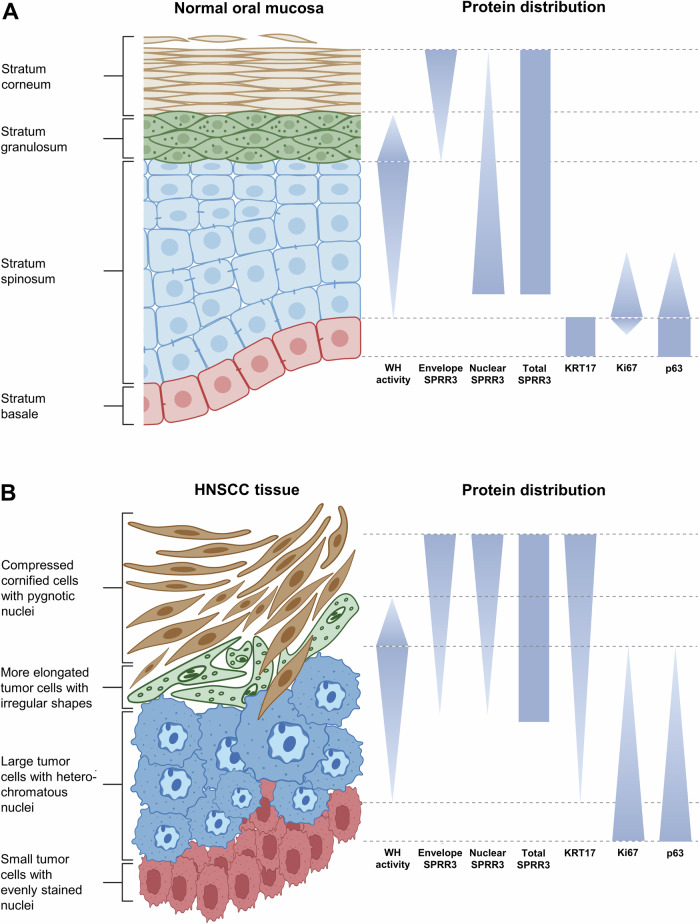
Architecture of oral mucosa and HNSCC tissue. Schematic visualization of cell layer organization in **A** normal oral mucosa and **B** well-differentiated areas of HNSCC tissue. Protein marker distribution of KRT17, SPRR3, p63, proliferation marker Ki67 and transcription factors of the wound-healing-like signaling zone (WH; e.g. C/EBPβ and c-JUN) is depicted. Please note that tumor tissue of individual patients varies strongly in the proportional representation of each displayed layer. Created with Biorender.com.

In well-differentiated tumors, the basal-like p63+/Ki67+ cell layer is located predominantly at the tumor-stroma interface, whereas in the undifferentiated tumor of P1 p63+/Ki67+ stem-like cells were highly abundant. P1 tissue displays small patches of single-cell cornification with a wound-healing-associated signaling zone consisting of individual cells surrounding SPRR3-positive cornifying cells. Thus, we conclude that the differentiation structure of normal mucosa is retained in HNSCCs, including undifferentiated tumors (Fig. 7).

### KRT17 is an early marker of HNSCC differentiation

Interestingly, RNA-seq analysis indicated KRT17 to be associated with HNSCC cell differentiation. We observed in various patients‘ tissue that KRT17 changed from a basal keratinocyte marker to an early differentiation marker preceding cornification in HNSCC. KRT17 is described as a stress-associated keratin in the skin and a marker of psoriasis [19]. It is plausible that tumor cells upregulate stress-associated signaling as oncogenic genetic alterations cause abnormalities in cell metabolism, protein folding, and other vital processes. In tumor tissue however, KRT17 is well-known as an oncoprotein that promotes proliferation, immune evasion, survival, and other malignant hallmarks [32]. KRT17 confers increased resistance to cisplatin in cervical cancer [33] and bladder cancer [34], and elevated expression of this gene was described to correlate with poor survival in oropharyngeal squamous cell carcinoma [35]. These observations contrast with our results, showing diminished tumor-initiating capacity of differentiated KRT17^high^ cells and an upregulation of this marker in proximity to cornified areas in the HNSCC tissue of distinct patients. It is possible that differentiated KRT17^high^ HNSCC cells are unable to repopulate the tumor but still facilitate oncopromoting functions. Moreover, tumors in advanced stages with higher tumor burden may contain more differentiated KRT17^high^ cells than smaller tumors due to more widespread necrosis. This would associate poor prognosis with high KRT17 levels despite the lack of a causal connection. However, our data is supported by previous reports suggesting KRT17 as differentiation marker in HNSCCs due to its spatial expression pattern [18, 35], and we are the first to present evidence on a functional level for an association of KRT17 with cellular differentiation and loss of malignancy. Interestingly, our data also suggest that premalignant mucosa reflects the tumor’s KRT17 expression pattern, with a much stronger abundance of this marker compared to normal mucosa. Thus, we propose KRT17 as a potential biomarker for early HNSCC detection.

### Differentiation therapies may be applicable in squamous cell carcinomas

Our study identifies distinct HNSCC cell states within the differentiation process, suggesting a rather hierarchical organization of long-term tumor repopulation. Tumor-initiating cell dynamics have been investigated previously in colon cancer (COCA) on a functional level. Using clonal marking experiments in serial transplantation in mice, researchers described a hierarchical organization of the colon TIC compartment with a subpopulation of long-term repopulating cells and tumor-transiently amplifying cells that lost self-renewal capacity and support tumor growth for a limited time frame [36]. This reflects the organization of normal colon regeneration with self-renewing LGR5+ stem cells and transiently amplifying cells to derive mature gut epithelial cells [37]. Moreover, another study revealed that colon cancer tissue is structured similarly to the normal colon epithelium with LGR5+ stem cells that give rise to KRT20+/LGR5− differentiated cells [38]. However, KRT20+/LGR5− cells can efficiently repopulate the LGR5+ stem-like compartment upon its depletion, which demonstrates that differentiation in COCA is not terminal [7]. In another tumor type, pancreatic ductal adenocarcinoma (PDAC), long-term tissue repopulation was found to be similar in benign and malignant tissue with a clonal succession of transient TICs [8], reflecting the transient de-differentiation of acinar cells to regenerate defects in the normal exocrine pancreas [39]. Thus, COCA and PDAC TICs display plasticity in their phenotypic and functional differentiation allowing tumors to avoid terminal differentiation by epigenetic mechanisms [25]. In contrast, our HNSCC differentiation model leads to cornification and loss of malignant behavior, so that differentiation therapies may be more efficient in this neoplasm than in other cancer types. Squamous cell carcinomas of different tissues of origin (e.g., lung or skin) may also be vulnerable to such treatment.

However, future research must be conducted to find an effective treatment regimen that is usable in clinics. Chemical activators of inflammatory signaling pathways and inhibitors of anti-inflammatory mechanisms might be suitable to eradicate long-term repopulating HNSCC cells. To our knowledge, the targeted differentiation of cancer cells was only achieved in neuroblastoma cells upon retinoid-induced ablation of their main oncogenic driver MYCN [40] but previously not in any type of carcinoma. The systemic application of retinoids has been investigated before to decrease the relapse of HNSCCs after surgery, providing negative results [41]. Possibly, targeted differentiation represents a strategy to reduce tumor relapse by injection of differentiating agents into the tumor site after surgery.

## Supplementary information

Supplementary Material

Supplementary tables

## Data Availability

All sequencing raw data are available on the NCBI Gene Expression Omnibus database under the GEO accession numbers GSE274251 for the ATAC-seq and GSE273699 for the RNA-seq analysis.
