# Supplementary material for: Mucosa-like differentiation of head and neck cancer cells is inducible and drives the epigenetic loss of cell malignancy
Source: Cell Death Dis. 2024 Oct 2;15(10):724. doi: 10.1038/s41419-024-07065-y (PMC11446932; doi:10.1038/s41419-024-07065-y)
Supplement: Supplementary file 1 — Supplementary Material [file 41419_2024_7065_MOESM1_ESM.pdf]

SUPPLEMENTARY MATERIAL

Supplementary Figure S1: HNSCC model of patient 2 (P2)

Supplementary Figure S2: Histology analysis of xenograft tumors in immunodeficient mice

Supplementary Figure S3: Signaling pathway activation upon cell adhesion

Supplementary Figure S4: Proteome analysis of the differentiation medium

Supplementary Figure S5: Accessibility of the *KRT17* locus in undifferentiated and differentiated HNSCC cells.

Supplementary Figure S6: Histology of original tumor tissue of patients 3-7

Supplementary Table S1: Patient characteristics

Supplementary Table S2: Taq information of the differential peak analysis of the ATAC-Seq

Supplementary Table S3: Genome peak distribution of the ATAC-Seq from patient 1 and patient 2

Supplementary Table S4: The differential peak analysis of the ATAC-Seq of patient 1

Supplementary Table S5: The differential peak analysis of the ATAC-Seq of patient 2

Supplementary Table S6: ATAC-Seq differential OCRs GO:BP patient 1

Supplementary Table S7: ATAC-Seq differential OCRs GO:BP patient 2

Supplementary Table S8: The differential expression analysis of the RNA-Seq of patient 1 diff. medium upregulated

Supplementary Table S9: The differential expression analysis of the RNA-Seq of patient 1 diff. medium downregulated

Supplementary Table S10: The differential expression analysis of the RNA-Seq of patient 2 diff. medium upregulated

Supplementary Table S11: The differential expression analysis of the RNA-Seq of patient 2 diff. medium downregulated

Supplementary Table S12: RNA-Seq diff. upregulated GO:BP patient 1

Supplementary Table S13: RNA-Seq diff. downregulated GO:BP patient 1

Supplementary Table S14: RNA-Seq diff. upregulated GO:BP patient 2

Supplementary Table S15: RNA-Seq diff. downregulated GO:BP patient 2

Supplementary Table S16: Proteome of the stem cell medium (SCM) identified using ESI-LC-MS

Supplementary Table S17: Proteome of the differentiation medium (CFM) identified using ESI-LC-MS

Supplementary Table S18: List of identified amino acids in differentiation medium (CFM) and stem cell medium (SCM) with their respective retention times and the m/z value used for quantitation

Supplementary Methods

Supplementary References

Please find Supplementary Tables S1-S18 in a separate document.

Supplementary Figures

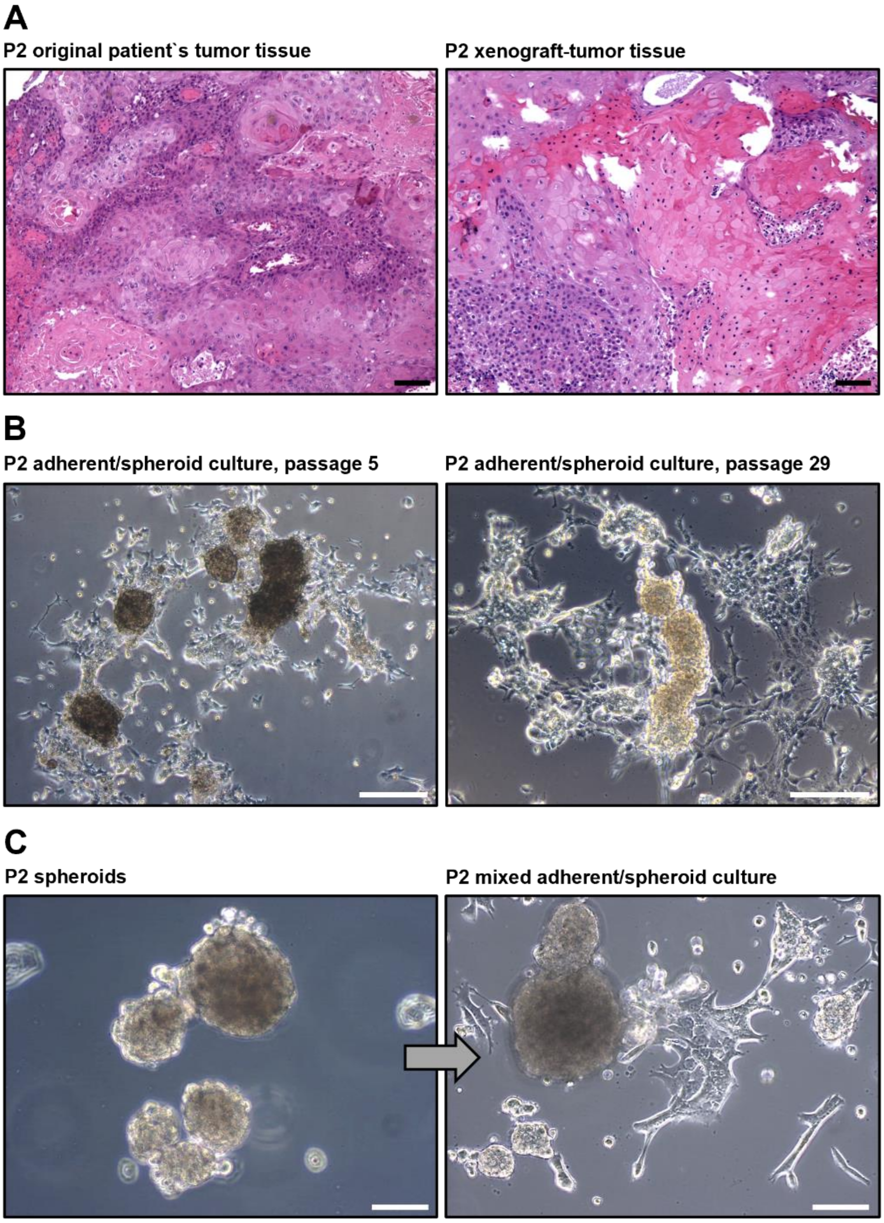

Supplementary Figure S1: HNSCC model of patient 2 (P2). (A) Original patient`s tumor and xenograft-tumor in immunodeficient NSG mice display similar histology; scale bars = 100 µm. **(B)** P2 cells from an adherent layer and give rise to spheroids that are gradually released into the culture medium; scale bars = 500 µm. **(C)** P2 spheroid cells can re-establish the mixed adherent/spheroid phenotype; scale bars = 100 µm.

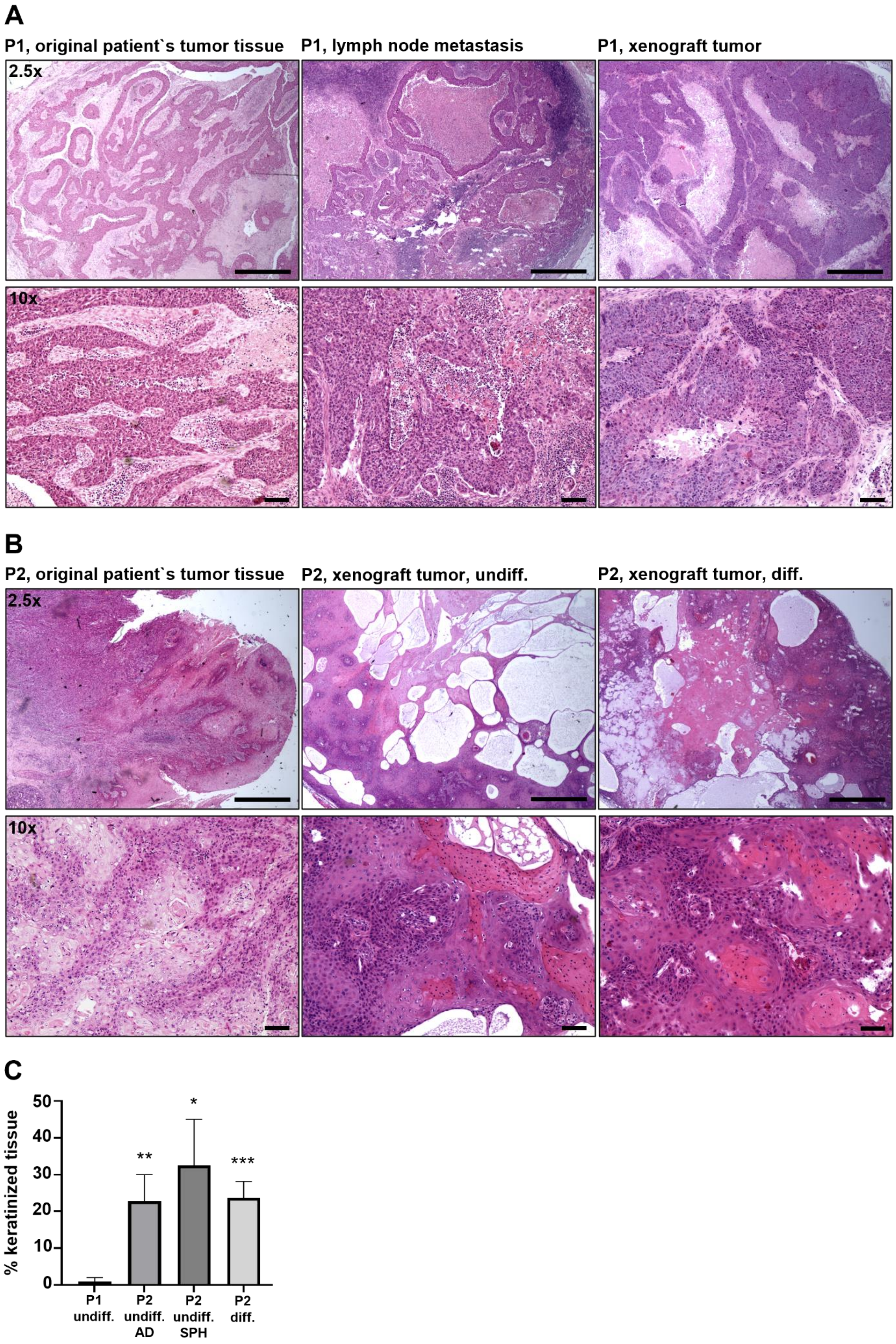

A
P1, original patient`s tumor tissue
P1, lymph node metastasis
P1, xenograft tumor
2.5x
10x
B
P2, original patient`s tumor tissue
P2, xenograft tumor, undiff.
P2, xenograft tumor, diff.
2.5x
10x
C
% keratinized tissue
50
40
30
20
10
0
**
*
***
P1 undiff.
P2 undiff. AD
P2 undiff. SPH
P2 diff.

Supplementary Figure S2: Histology analysis of xenograft tumors in immunodeficient mice. (A) HE-stained sections of P1 xenograft tumors display a cystic growth pattern with large areas of necrosis consistent with lymph node metastases of P1; scale bars = 1 mm upper panel, 100 µm lower panel. **(B)** P2 xenograft tumors initiated by differentiated and undifferentiated cell cultures show a similar cystic histology; scale bars = 1 mm. **(C)** Proportional quantification of keratinization in HNSCC xenograft tumor tissue of P1 and P2 in immunodeficient mice by histopathology analysis. Tumors initiated by all P2 populations were significantly higher keratinized than P1 tumors induced by undifferentiated cells: *p < 0.01, **p < 0.005, ***p < 0.0005; determined by student\`s t-test; n = 9 for P1 and n = 12 for each P2 population. Differentiation medium-treated (CFM) cells of P1 did not induce any xenograft tumors in mice in this experiment. All differences among P2 tumor populations were not significant. Significance levels were determined by student\`s t-test; AD = adherent; SPH = spheroid.

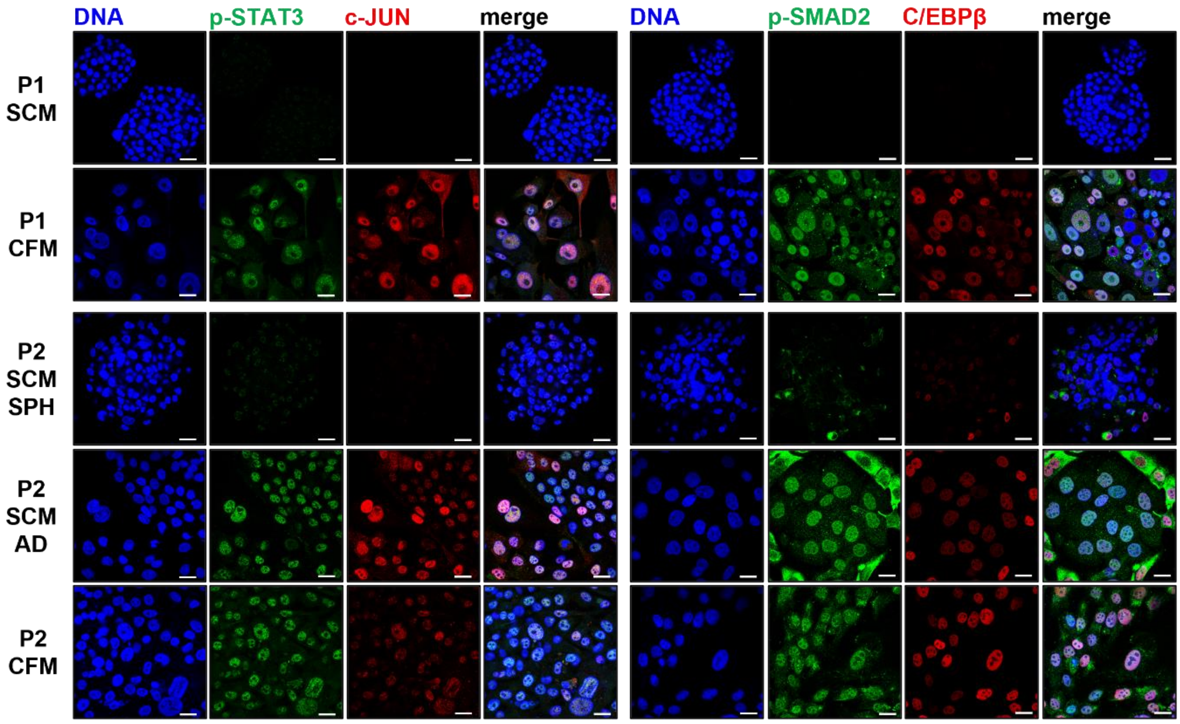

Supplementary Figure S3: Signaling pathway activation upon cell adhesion. IF staining reveals nuclear localization of TGFβ/SMAD2, c-JUN/AP-1, JAK/STAT3, and C/EBPβ in adherent cells of P1 and P2; scale bars = 10 µm.

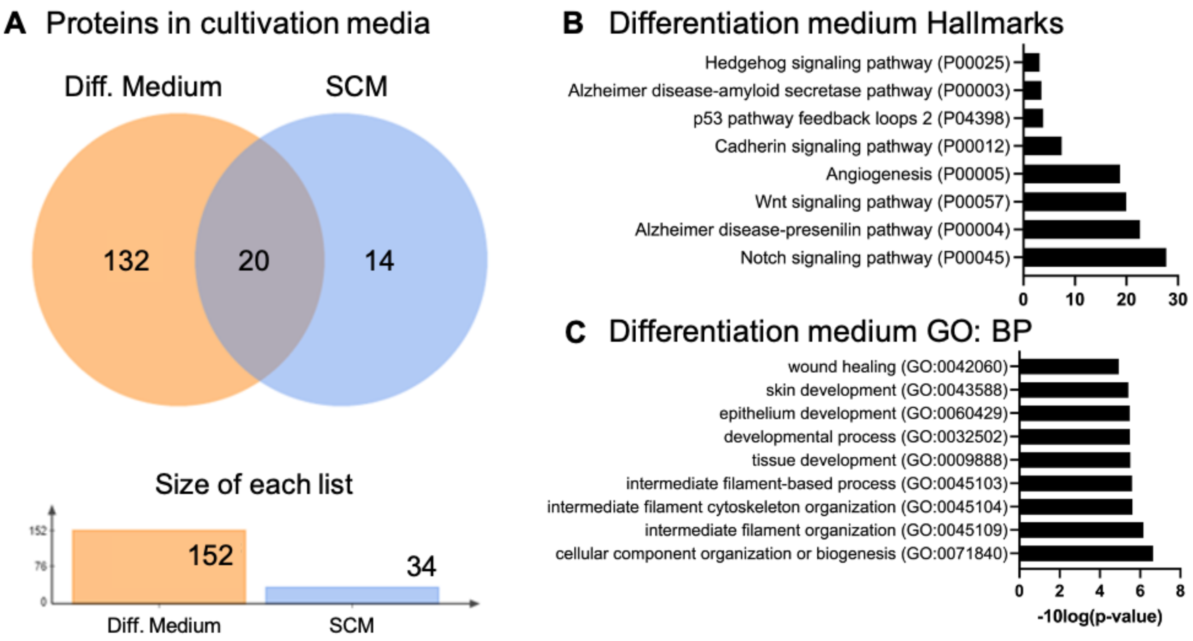

Supplementary Figure S4: Proteome analysis of the differentiation medium. (A) All identified proteins in the differentiating medium (orange) and control medium SCM (blue). A total of 152 proteins were found in the differentiation medium, 20 of which are also included in the control medium SCM. In SCM, 34 proteins were identified. **(B)** Enrichment analysis of the proteins contained in differentiation media revealed significant Hallmark pathways. **(C)** Enrichment analysis of the proteins contained in differentiation media revealed significant Gene Ontology Biological Process pathways.

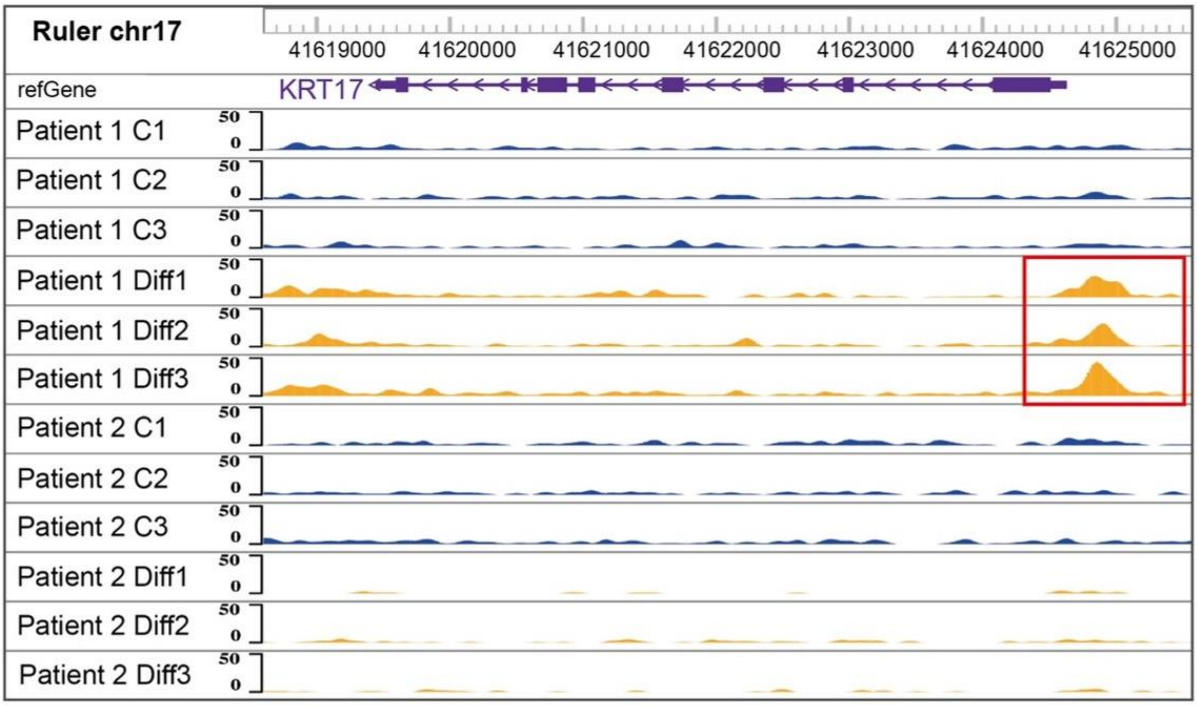

Supplementary Figure S5: Accessibility of the *KRT17* locus in undifferentiated and differentiated HNSCC cells. *KRT17* was only well accessible in highly differentiation sensitive cultures of P1. Displayed are three independent samples of differentiated and undifferentiated cells of P1 and P2.

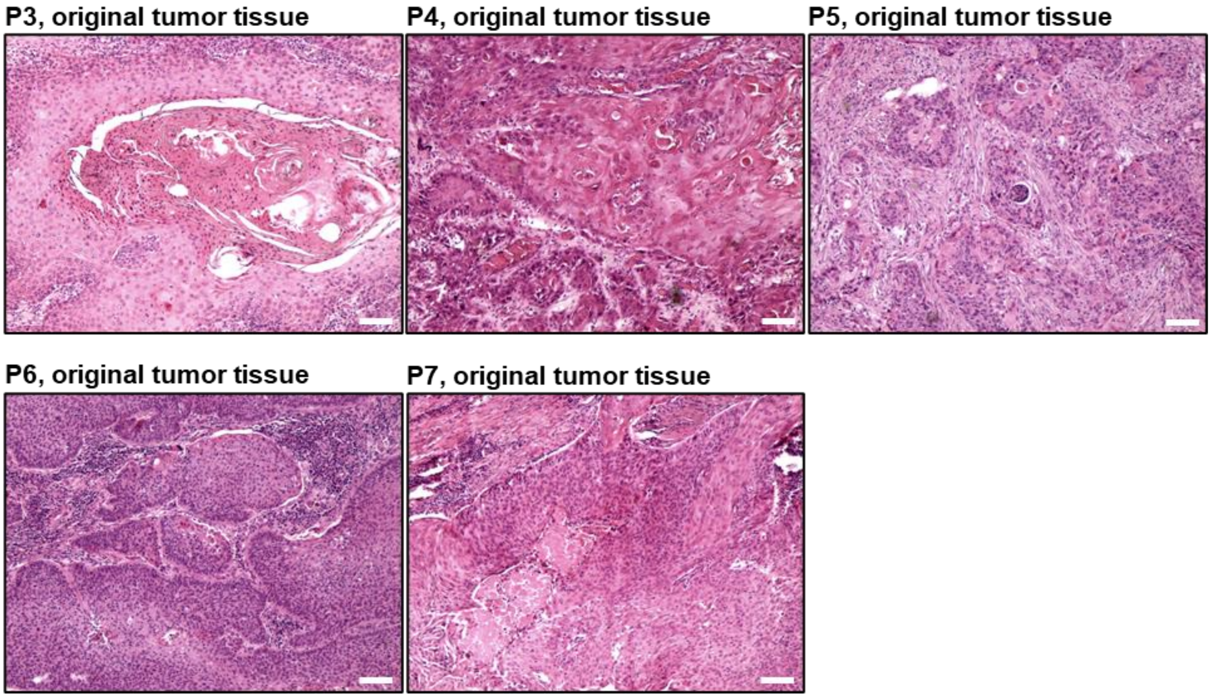

Supplementary Figure S6: Histology of original tumor tissue of patients 3-7. Hematoxylin and eosin staining; scale bars = 100 µm.

Supplementary Methods

Drug treatment of HNSCC cells

Small tumor spheroids (<100 µm diameter) were seeded in the appropriate medium in 12-well or 24-well cell culture dishes (STARLAB, Hamburg, Germany) containing 12 mm or 18 mm #1 cover slips (Carl Roth, Karlsruhe, Germany). Adherent cells were seeded at a density of 1-5x10^4 cells per well. Cells were treated with small molecules or recombinant human cytokines directly after seeding and incubated for 3 to 5 days. After that, cells were washed with phosphate buffered saline (PBS, Capricorn Scientific) and fixed with 4% PFA in PBS for 25 min. Cell populations were treated with EGF, IL1α, IL1β, IL6, IL17A, IL19, IL20, IL22, IL24, TGFβ, OSM, TNFα (30ng/ml; all PeproTec, Hamburg, Germany) or Fetuin-B (30ng/ml; FETUB, NP_055190, R&D

Systems/Bio-Techne, Abingdon, United Kingdom). Cytokine treatments were performed using SCM without hydrocortisone.

Antibodies used in indirect immunofluorescence analysis of cells and tissue

Primary antibodies: mouse-anti-human C/EBPβ (1:100, H-7, sc-7962), mouse-anti-human c-JUN (1:100, G-4, sc-74543), mouse-anti-human esophagin (SPRR3, 1:100, E-6, sc-514844), mouse-anti-human KRT17 (1:100, E-4, sc-393002), mouse-anti-human p63 (1:100, D-9, sc-25268), mouse-anti-human SMAD2/3 (1:100, A-3, sc-398844, all Santa Cruz Biotechology, Dallas, TX, United States), rabbit-anti-human Ki67 (1:200, SP6, ab16667, Abcam), rabbit-anti-human SPRR3 (1:200, NBP2-13374, Novus Biologicals/Bio-Techne, Abingdon, United Kingdom), rabbit-anti-human phospho-STAT3-Y705 (1:200, A16431, Antibodies Online, Aachen, Germany), rabbit-anti-human phospho-SMAD2-S465/S467 (1:50, E8F3R, 18338T, Cell Signaling Technologies), and guinea pig-anti-human KRT17; secondary antibodies: goat-anti-mouse-IgG-Alexa Fluor-555 (1:400, A21422), donkey-anti-rabbit-IgG-Alexa Fluor-488 (1:400, A11008), and goat-anti-guinea pig-IgG-Alexa Fluor-647 (1:400, A21450, all Thermo Fisher Scientific, Waltham, MA, United States).

RNA Isolation

Cells were cultured for eight days. Total RNA was isolated from cell culture (n=3) using innuPREP DNA/RNA Mini Kit (Analytik Jena, Jena, Germany) as recommended by the manufacturer's protocol. RNA quality was determined with a BioDrop Duo+ spectral photometer (Biochrom, Holliston, USA).

RNA-seq Library Preparation and Sequencing

PolyA-selected libraries were prepared from 200 ng of total RNA using QuantSeq 3′mRNA-Seq Library Prep Kit FWD for Illumina (Lexogen), according to the manufacturer's instructions. Size distribution and quality of the libraries were assessed by fragment analyzer (Agilent) and final libraries were sequenced in 75 bp single-end mode on a NextSeq2000 with P3 chemistry.

Analysis of RNA-seq Data

RNA-seq analysis was performed with technical replicates n=3. The single-end reads were mapped to the human transcriptome (RefSeq Transcripts GRCh38; downloaded from NCBI) and quantified using Kallisto v0.44.0 (42). Estimated counts were statistically analyzed using edgeR v3.28.1 (43). The fold-change gene expression values were calculated in pairwise comparisons between the undifferentiated cancer cells as the control and differentiated cancer cells as treated. Genes were considered differentially expressed if their p-value was <0.05. Gene ontology analysis was processed using the GO web tool (44-46) with the human annotations applying Fisher's exact test. The GO annotation was visualized using GraphPad PRISM v9.0 (GraphPad Software, Inc). Gene set enrichment and leading-edge analyses were generated with GSEA software v4.2.1 (47, 48) with 1000 permutation numbers and a nominal p-value <0.05. The gene set enrichment map was generated using Cytoscape v3.9.1, with a cut-off value of p 0.01 (49).

ATAC-seq Library Preparation and Sequencing

ATAC-seq analysis was performed with technical replicates (n=3). ATAC-seq was performed according to Buenrostro *et al.* (50) with harvested P1 and P2 cells, including isolation of intact nuclei and tagging with Tn5 transposase. After cell preparation, transposition reaction, library preparation, and purification, the quality of libraries was assessed using an Agilent Bioanalyzer High Sensitivity DNA Analysis kit. Sequencing was performed first on an Illumina MiSeq system in paired-end mode 2 x 50 bp and finally on the Illumina NextSeq 2000 system in paired-end mode 2 x 50 bp with P3 chemistry.

Analysis of ATAC-seq Data

The ATAC-seq reads of each sample were mapped to the human reference genome GRCh38 using Bowtie2 v2.5.0 with default settings (51). Samtools v1.16.1 was used for formatting, quality filtering, and removing duplicates (52) to guarantee unique mapping; the MAPQ (mapping quality, 0=non-unique, >10 probably unique) was set to a sophisticated value of 30. After examining each replicate separately, replicates of respected groups were combined to maximize the peak calling strength using the samtools merge function. MACS3 v3.0.0b1 was used for peak calling with the parameters "–nomodel –nolambda –keep-dup auto –call summits" the peaks were filtered by a q-value cutoff of 0.05 (53). The sequences of peaks from 250 bp upstream and 250 bp downstream of the peaks were extracted with R v4.2.2 and Bedtools v2.30.0 (54) (R-Citation). In R v4.2.2 GenomicRanges v1.42.0 linked the ATAC-Seq peaks with the nearest genes. The promoter was defined as the region within 2 kb of the reference transcript start site (55). Enriched motifs in the peak sequences were analyzed with MEME-ChIP v5.5.1 and default settings (56). Gene ontology analysis of

the identified motifs was done with GOMo v5.5.1 (57). The differential peak analysis of the ATAC-seq was performed using HOMER2 v4.11 with a replicate FDR cutoff of 0.05 for peak identification calculated by DESeq2 v1.24.0 (58). The genomic annotation of the peaks was determined using ChIPSeeker v1.34.1 (59, 60). The genomic distribution of peaks was visualized using Adobe Illustrator 2023 (Adobe Inc., San Jose, USA).

Cornification Assay

Cornification was quantified based on a previous protocol to isolate cornified envelopes (61). Triplicates of P1 and P2 cells were seeded into 6-well plates in SCM or CFM until cells were 80-90% confluent. Cells were washed with 1x PBS, detached using Accutase (Capricorn Scientific, Ebsdorfergrund, Germany), and stained with trypan blue (Sigma Aldrich), followed by a count of live and dead cells using a Neubauer Chamber. The cells were washed in 1x PBS and subsequently mixed with 100 µL 4% SDS (Carl Roth GmbH, Karlsruhe, Germany) and 2% beta-Mercaptoethanol (Merck, Darmstadt, Germany) in PBS. The suspension is cooked at 95°C for 5-10 min. Lastly the cornified envelopes were counted using a Neubauer Chamber.

Proliferation Assay

Triplicates of P1 and P2 cells ($5x10^4$) were seeded into 12-well plate in SCM or CFM. After 5 days of incubation at 37°C with 5% CO_2 cells were washed with 1x PBS and detached using Accutase (Capricorn Scientific, Ebsdorfergrund, Germany). The cells were manually counted using a Neubauer Chamber. After that, cells were washed in 1x PBS, resuspended in culture medium and further incubated at the same conditions.

The growth curves were generated over a period of 15 days, with repeated counting every 5 days.

GC-MS Analysis

A TraceGC gas chromatograph was utilized to analyze 1 µL sample volumes. The chromatograph was connected to a PolarisQ ion trap mass spectrometer, both made by Thermo Finnigan in Dreieich, Germany. Metabolites were identified using purified standards, the NIST 2005 database (NIST, Gaithersburg, MD, USA) (NistDatabase, 2005). All identified metabolites were matched with the references by mass spectral data and chromatographic retention time. The Xcalibur 1.4 software (Thermo Finnigan, Dreieich, Germany) was used to quantify the peak areas of the chosen metabolites automatically. The peak areas were used to derive relative response ratios, which were then normalized by the internal standard ribitol and the dry mass of the sample.

Protein Isolation/In-Solution Digest

The samples were resuspended in 100 µL of 100 mM ammonium bicarbonate (Ambic, Sigma Aldrich), 100 µL trifluoroethanol (TFE, Fluka Biochemica), 5 µL of 200 mM dithiothreitol (DTT, Sigma Aldrich) and incubated at 60°C for 60 min at low rpm. Then, 20 µL of 200 mM iodoacetamide (IAA, Sigma Aldrich) was added, and samples were incubated in the dark for 90 min. Hereafter, the step was repeated with DTT, and the solution was resuspended in Ambic at a ratio of 1:1. Samples were filled up to a total volume of 990 µL with MilliQ, added 10 µL Trypsin Gold (Promega, (Promega, Madison, Wisconsin, United States) and incubated overnight at 37 °C. Samples were

centrifuged at 12000 rpm for 2 min to separate peptides from cellular residues. The supernatant containing the peptides was diluted 1:1 with Solution A. The purification of the proteins was carried out via the Sep-PAK C18 Vac Cartridges (Waters, Milford, Massachusetts, United States) using solutions A and B (A: 98 % MilliQ (MS Grade), 0.2 % acetonitrile (MS Grade), 0.1 % TFA; B: 35 % MilliQ (MS Grade), 65 % acetonitrile (MS Grade), 0.1 % TFA). The sample solution was diluted 1:1 with solution A while the columns were equilibrated with solution B and washed with solution A. Then, the samples were applied, washed with solution A, and eluted twice with 50 µL solution B. The eluate was evaporated in a SpeedVac, resolubilized in 12 µL solution A, and diluted to a protein concentration of 1 µg/µL. The samples were transferred into vials and injected into the NanoLC-ESI MS/MS. 12

Nano-LC-ESI MS/MS Analysis

The measurement was performed on a chromatograph Dionex Ultimate 3000 RSLC connected to the Orbitrap ESI-MS Q-Exactive-Plus. Peptide trapping on a precolumn Acclaim PepMapTM 100 column (5 mm x 300 µm) was followed by the peptide separation on an Acclaim PepMapTM column (25 cm x 75 µm) (all Thermo Fisher Scientific, Waltham, Massachusetts, United States). Mobile phase gradient elution of solution A (0.1% formic acid in H2O) mixed with solution B (0.08% formic acid in 80% acetonitrile) was performed as follows: 0-5 min at 4% B, 120 min at 30% B, 7 min at 50% B, 8 min at 95% B, 5 min at 4% with a flow rate of 0.3 µm/ml. The ESI brings the positively charged ions into the gas phase with a 1.8 to 2 kV voltage. The orbitrap instrument was operated in Full MS/data-dependent MS2 (Top10) mode. The scan range for ions was 300 to 1600 m/z at a resolution of 70000 full widths at half maximum using a maximum injection time (ITmax) of 64 ms and an automatic gain control (AGC)

target 3e6. Ions with a charge state of z=2 were filtered at an isolation width of m/z 1.6 for the HCD fragmentation at 28% collision energy. In addition, a charge exclusion was selected to exclude 1-charged and 8-charged ions from the measurement. The MS2 ions were scanned at 17500 widths at half maximum (ITmax=120ms, AGC=2e5). The NanoLC-ESI MS/MS instrument was controlled by Xcalibur 4.4.16.14 and Tune Tune 2.11 QF2 Build 3007.

Protein Database Search

The human Database of UniProt (taxID: 9606) was used via Proteome Discoverer 3.0 to identify proteins. For the tryptic digestion, two missed cleavages were allowed with a precursor mass tolerance of 10 ppm and a fragment mass tolerance of 0.02 Da. Dynamic methionine oxidation and protein N-term acetylation were defined as dynamic modifications, while carbamidomethylation was defined as a static modification. At least one peptide per protein and a strict FDR of 1% was required for positive protein hits. The percolator and reverse decoy database were used for the q-value validation of spectral matches.

GC-MS Analysis

A TraceGC gas chromatograph was utilized to analyze 1 µL sample volumes. The chromatograph was connected to a PolarisQ ion trap mass spectrometer, both made by Thermo Finnigan in Dreieich, Germany. Metabolites were identified using purified standards, the NIST 2005 database (NIST, Gaithersburg, MD, USA) (NistDatabase, 2005). All identified metabolites were matched with the references by mass spectral data and chromatographic retention time. The Xcalibur

1.4 software (Thermo Finnigan, Dreieich, Germany) was used to quantify the peak areas of the chosen metabolites automatically. The peak areas were used to derive relative response ratios, which were then normalized by the internal standard ribitol and the dry mass of the sample.

Protein Isolation/In-Solution Digest

The samples were resuspended in 100 µL of 100 mM ammonium bicarbonate (Ambic, Sigma Aldrich), 100 µL trifluoroethanol (TFE, Fluka Biochemica), 5 µL of 200 mM dithiothreitol (DTT, Sigma Aldrich) and incubated at 60°C for 60 min at low rpm. Then, 20 µL of 200 mM iodoacetamide (IAA, Sigma Aldrich) was added, and samples were incubated in the dark for 90 min. Hereafter, the step was repeated with DTT, and the solution was resuspended in Ambic at a ratio of 1:1. Samples were filled up to a total volume of 990 µL with MilliQ, added 10 µL Trypsin Gold (Promega, (Promega, Madison, Wisconsin, United States) and incubated overnight at 37 °C. Samples were centrifuged at 12000 rpm for 2 min to separate peptides from cellular residues. The supernatant containing the peptides was diluted 1:1 with Solution A. The purification of the proteins was carried out via the Sep-PAK C18 Vac Cartridges (Waters, Milford, Massachusetts, United States) using solutions A and B (A: 98 % MilliQ (MS Grade), 0.2 % acetonitrile (MS Grade), 0.1 % TFA; B: 35 % MilliQ (MS Grade), 65 % acetonitrile (MS Grade), 0.1 % TFA). The sample solution was diluted 1:1 with solution A while the columns were equilibrated with solution B and washed with solution A. Then, the samples were applied, washed with solution A, and eluted twice with 50 µL solution B. The eluate was evaporated in a SpeedVac, resolubilized in 12 µL solution A, and diluted to a protein concentration of 1 µg/µL. The samples were transferred into vials and injected into the NanoLC-ESI MS/MS.

Nano-LC-ESI MS/MS Analysis

The measurement was performed on a chromatograph Dionex Ultimate 3000 RSLC connected to the Orbitrap ESI-MS Q-Exactive-Plus. Peptide trapping on a precolumn Acclaim PepMapTM 100 column (5 mm x 300 µm) was followed by the peptide separation on an Acclaim PepMapTM column (25 cm x 75 µm) (all Thermo Fisher Scientific, Waltham, Massachusetts, United States). Mobile phase gradient elution of solution A (0.1% formic acid in H2O) mixed with solution B (0.08% formic acid in 80% acetonitrile) was performed as follows: 0-5 min at 4% B, 120 min at 30% B, 7 min at 50% B, 8 min at 95% B, 5 min at 4% with a flow rate of 0.3 µm/ml. The ESI brings the positively charged ions into the gas phase with a 1.8 to 2 kV voltage. The orbitrap instrument was operated in Full MS/data-dependent MS2 (Top10) mode. The scan range for ions was 300 to 1600 m/z at a resolution of 70000 full widths at half maximum using a maximum injection time (ITmax) of 64 ms and an automatic gain control (AGC) target 3e6. Ions with a charge state of z=2 were filtered at an isolation width of m/z 1.6 for the HCD fragmentation at 28% collision energy. In addition, a charge exclusion was selected to exclude 1-charged and 8-charged ions from the measurement. The MS2 ions were scanned at 17500 widths at half maximum (ITmax=120ms, AGC=2e5). The NanoLC-ESI MS/MS instrument was controlled by Xcalibur 4.4.16.14 and Tune Tune 2.11 QF2 Build 3007.

Protein Database Search

The human Database of UniProt (taxID: 9606) was used via Proteome Discoverer 3.0 to identify proteins. For the tryptic digestion, two missed cleavages were allowed with a precursor mass tolerance of 10 ppm and a fragment mass tolerance of 0.02 Da. Dynamic methionine oxidation and protein N-term acetylation were defined as dynamic

modifications, while carbamidomethylation was defined as a static modification. At least one peptide per protein and a strict FDR of 1% was required for positive protein hits. The percolator and reverse decoy database were used for the q-value validation of spectral matches.

Supplementary References

42. Bray NL, Pimentel H, Melsted P, Pachter L. Near-optimal probabilistic RNA-seq quantification. Nature biotechnology. 2016;34(5):525-7.

43. McCarthy DJ, Chen Y, Smyth GK. Differential expression analysis of multifactor RNA-Seq experiments with respect to biological variation. Nucleic acids research. 2012;40(10):4288-97.

44. Ashburner M, Ball CA, Blake JA, Botstein D, Butler H, Cherry JM, et al. Gene ontology: tool for the unification of biology. The Gene Ontology Consortium. Nature genetics. 2000;25(1):25-9.

45. Gene Ontology C. The Gene Ontology resource: enriching a GOld mine. Nucleic acids research. 2021;49(D1):D325-D34.

46. Mi H, Muruganujan A, Ebert D, Huang X, Thomas PD. PANTHER version 14: more genomes, a new PANTHER GO-slim and improvements in enrichment analysis tools. Nucleic acids research. 2019;47(D1):D419-D26.

47. Subramanian A, Tamayo P, Mootha VK, Mukherjee S, Ebert BL, Gillette MA, et al. Gene set enrichment analysis: a knowledge-based approach for interpreting genome-wide expression profiles. Proceedings of the National Academy of Sciences of the United States of America. 2005;102(43):15545-50.

48. Mootha VK, Lindgren CM, Eriksson KF, Subramanian A, Sihag S, Lehar J, et al. PGC-1alpha-responsive genes involved in oxidative phosphorylation are coordinately downregulated in human diabetes. Nature genetics. 2003;34(3):267-73.

49. Shannon P, Markiel A, Ozier O, Baliga NS, Wang JT, Ramage D, et al. Cytoscape: a software environment for integrated models of biomolecular interaction networks. Genome research. 2003;13(11):2498-504.

50. Buenrostro JD, Wu B, Chang HY, Greenleaf WJ. ATAC-seq: A Method for Assaying Chromatin Accessibility Genome-Wide. Current protocols in molecular biology. 2015;109:21 9 1- 9 9.

51. Langmead B, Salzberg SL. Fast gapped-read alignment with Bowtie 2. Nature methods. 2012;9(4):357-9.

52. Li H, Handsaker B, Wysoker A, Fennell T, Ruan J, Homer N, et al. The Sequence Alignment/Map format and SAMtools. Bioinformatics. 2009;25(16):2078-9.

53. Zhang Y, Liu T, Meyer CA, Eeckhoute J, Johnson DS, Bernstein BE, et al. Model-based analysis of ChIP-Seq (MACS). Genome biology. 2008;9(9):R137.

54. Quinlan AR, Hall IM. BEDTools: a flexible suite of utilities for comparing genomic features. Bioinformatics. 2010;26(6):841-2.

55. Lawrence M, Huber W, Pages H, Aboyoun P, Carlson M, Gentleman R, et al. Software for computing and annotating genomic ranges. PLoS computational biology. 2013;9(8):e1003118.

56. Bailey TL, Johnson J, Grant CE, Noble WS. The MEME Suite. Nucleic acids research. 2015;43(W1):W39-49.

57. Buske FA, Boden M, Bauer DC, Bailey TL. Assigning roles to DNA regulatory motifs using comparative genomics. Bioinformatics. 2010;26(7):860-6.

58. Heinz S, Benner C, Spann N, Bertolino E, Lin YC, Laslo P, et al. Simple combinations of lineage-determining transcription factors prime cis-regulatory elements required for macrophage and B cell identities. Molecular cell. 2010;38(4):576-89.

59. Yu G, Wang LG, He QY. ChIPseeker: an R/Bioconductor package for ChIP peak annotation, comparison and visualization. Bioinformatics. 2015;31(14):2382-3.

60. Wang Q, Li M, Wu T, Zhan L, Li L, Chen M, et al. Exploring Epigenomic Datasets by ChIPseeker. Current protocols. 2022;2(10):e585.

61. Jarnik M, Simon MN, Steven AC. Cornified cell envelope assembly: a model based on electron microscopic determinations of thickness and projected density. Journal of cell science. 1998;111 (Pt 8):1051-60.
